# Human Neural Stem Cell Biodistribution and Predicted Tumor Coverage by a Diffusible Therapeutic in a Mouse Glioma Model

**DOI:** 10.1002/sctm.16-0397

**Published:** 2017-05-08

**Authors:** Michael E. Barish, Kelsey Herrmann, Yang Tang, Siranush Argalian Herculian, Marianne Metz, Soraya Aramburo, Revathiswari Tirughana, Margarita Gutova, Alexander Annala, Rex A. Moats, Leanne Goldstein, Russell C. Rockne, Jennifer Gutierrez, Christine E. Brown, Lucy Ghoda, Karen S. Aboody

**Affiliations:** ^1^ Department of Developmental & Stem Cell Biology, City of Hope Beckman Research Institute and Medical Center Duarte California USA; ^2^ Department of Radiology, University of Southern California Los Angeles California USA; ^3^ Department of Pathology, Keck School of Medicine, University of Southern California Los Angeles California USA; ^4^ Department of Biomedical Engineering, Viterbi School of Engineering University of Southern California Los Angeles California USA; ^5^ Department of Information Sciences, City of Hope Beckman Research Institute and Medical Center Duarte California USA; ^6^ Department of Hematology/HCT, City of Hope Beckman Research Institute and Medical Center Duarte California USA; ^7^ Department of Immuno‐Oncology, City of Hope Beckman Research Institute and Medical Center Duarte California USA; ^8^ Department of Division of Neurosurgery City of Hope Beckman Research Institute and Medical Center Duarte California USA

**Keywords:** Neural stem cells, Biodistribution, Mouse model, Anticancer therapy, Diffusible therapeutic, Glioblastoma, Glioma

## Abstract

Engineered neural stem cells (NSCs) intrinsically migrating to brain tumors offer a promising mechanism for local therapeutic delivery. However, difficulties in quantitative assessments of NSC migration and in estimates of tumor coverage by diffusible therapeutics have impeded development and refinement of NSC‐based therapies. To address this need, we developed techniques by which conventional serial‐sectioned formalin‐fixed paraffin‐embedded (FFPE) brains can be analyzed in their entirety across multiple test animals. We considered a conventional human glioblastoma model: U251 glioma cells orthotopically engrafted in immunodeficient mice receiving intracerebral (*i.c*.) or intravenous (*i.v*.) administrations of NSCs expressing a diffusible enzyme to locally catalyze chemotherapeutic formation. NSC migration to tumor sites was dose‐dependent, reaching 50%–60% of total administered NSCs for the i.c route and 1.5% for the *i.v*. route. Curiously, the most efficient NSC homing was seen with smaller NSC doses, implying existence of rate‐limiting process active during administration and/or migration. Predicted tumor exposure to a diffusing therapeutic (assuming a 50 µm radius of action) could reach greater than 50% of the entire tumor volume for *i.c*. and 25% for *i.v*. administration. Within individual sections, coverage of tumor area could be as high as 100% for *i.c*. and 70% for *i.v*. routes. Greater estimated therapeutic coverage was observed for larger tumors and for larger tumor regions in individual sections. Overall, we have demonstrated a framework within which investigators may rationally evaluate NSC migration to, and integration into, brain tumors, and therefore enhance understanding of mechanisms that both promote and limit this therapeutic modality. Stem Cells Translational Medicine *2017;6:1522–1532*

**Significance Statement**

Neural stem cells (NSCs) intrinsically migrate to sites of brain tumors, and engineered NSCs offer a promising mechanism for local delivery of therapeutic agents. While many groups have observed that therapeutically modified NSCs migrate selectively to glioma foci, quantitative assessments of NSC migration efficiency and local distribution at tumor sites, as well as tumor coverage estimated for the therapeutics delivered by these NSCs, have been difficult to perform. In this article, we present a quantitative analysis of immunostained serially sectioned formalin‐fixed paraffin‐embedded (FFPE) brain tissue across multiple test animals, thereby providing a paradigm facilitating optimization of this and other cell‐based therapies.

## Introduction

Neural stem cells (NSCs) intrinsically migrate to sites of brain tumors, attracted and guided by soluble factors, extracellular matrices and intrinsic brain architecture 1, 2, 3, 4, 5, 6, 7, 8, 9, 10, 11, 12, 13, 14, 15, 16, 17. This migration can be quite rapid; NSCs injected into the hemisphere contralateral to a previously engrafted tumor can cross the corpus callosum to the tumor site in less than one hour 18. Endogenous NSCs intrinsically suppress tumor growth 3, 5, 8, 19, 20 and when introduced exogenously, they offer a mechanism for targeted delivery of therapeutics to invasive brain tumors including glioblastoma (GBM) 1, 2, 21, 22, 23, 24, 25, 26, 27, 28, 29, 30, 31.

While many groups have observed that therapeutically‐modified NSCs migrate selectively to glioma foci in rodent models following intracerebral (*i.c*.) or intravenous (*i.v*.) administration 23, 32, quantitative assessments of NSC migration efficiency and local distribution at tumor sites, as well as tumor coverage by therapeutics delivered by these NSCs, have been difficult to perform. This limitation has restricted evaluation of alternative delivery strategies and therapeutic payloads. To further refinement of future NSC‐based therapies and those under development 33, 34, 35, 36, 37, 38, 39, 40, 41, 42, we established techniques by which immunostained serially‐sectioned formalin‐fixed paraffin‐embedded (FFPE) brain tissue can be used for quantitative analyses across multiple test animals.

Here, as part of the preclinical IND‐enabling studies for an NSC‐based therapy for glioblastoma, we evaluated distributions of NSCs in and around tumors in an orthotopic xenograft model (U251 human glioma cells in immunodeficient mice) offering engraftment and growth characteristics sufficiently consistent to generate the reproducible measurements required for a quantitative study, and estimated the percentages of tumor volume that would exposed to diffusible therapeutic agents produced by the NSCs. Depending on the specific strategy, this could be a diffusible prodrug‐activating enzyme 1, 43, 44 an oncolytic virus 29, 45, or an antibody 27. These measurements are important for assessing potential clinical advantages and disadvantages of different NSC administration routes (intracerebral vs. intravenous) and regimens (single vs. multiple doses), and identifying barriers to optimal implementation of these therapies. These approaches to quantitative evaluation thus provide a paradigm aiding evaluation of alternative therapeutic designs and facilitating optimization of this and other cell‐based therapies 46.

## Materials and Methods

### Glioma‐Bearing Mice for In Vivo Studies

All animal studies were performed under approved City of Hope (IACUC #04011) and CHLA (IACUC #285) protocols. Host animals for this study were carboxylesterase (CE)‐deficient SCID mice (*Es1^e^*/SCID) 47, having plasma CE concentrations consistent with human levels of circulating CE 44 and better reproducing human conditions for the enzyme/prodrug therapy being investigated (as wild type mice have high plasma CE levels). *Es1^e^*/SCID mice were bred at both St. Jude and City of Hope Animal Breeding Facilities.

Human U251 glioma cells were lentivirus‐transduced to express eGFP and firefly luciferase (ffLuc) under control of a common EF‐1α promoter (U251T.eGFP.ffLuc) and grown in DMEM (Irvine Scientific; Santa Ana, CA; http://www.irvinesci.com/) supplemented with 10% FCS, 2 mM L‐glutamine, and 25 mM HEPES. Adult *Es1^e^*/SCID mice received stereotactic frontal lobe injections under ketamine/xylazine anesthesia of U251T.eGFP.ffluc glioma cells (2 × 10^5^ cells) in 2 µl phosphate buffered saline (PBS) 1 mm rostral and 2 mm right of the bregma, at an initial depth of 2.5 mm followed by retraction to 2.25 and 2.0 mm, with 0.667 μl injected at each depth to maximize tumor‐host tissue interactions.

### Preparation of NSCs

Immortalized human HB1.F3.CD NSCs 48, 49 stably expressing cytosine deaminase 50 (HB1.F3.CD clone 21) were cultured in T‐175 tissue culture flasks in DMEM (Invitrogen, 10313‐021; Carlsbad CA; www.thermofisher.com/us/en/home/brands/invitrogen.html) supplemented with 10% heat‐inactivated FBS (Hyclone, SH30070.03; Logan, Utah; www.gelifesciences.com/webapp/wcs/stores/servlet/catalog/en/GELifeSciences-us/brands/hyclone/) and 2 mM L‐glutamine (Invitrogen, 25030‐081). Of clinical relevance, we used the same HB1.F3.CD (clone 21) NSCs that were used in our initial first‐in‐human NSC glioma study 51.

These NSCs were further engineered for high‐transient expression of a modified human CE by transduction with replication‐deficient adenoviral construct hCE1m6 44, 52 (here referred to as hCE1m6‐NSCs). Secreted hCE1m6 CE catalyzes local conversion of the systemically administered prodrug CPT‐11 (irinotecan) to the potent topoisomerase‐1 inhibitor and chemotherapeutic agent SN‐38.

To permit in vivo tracking by MR imaging in other studies, NSCs were also loaded with superparamagnetic iron oxide nanoparticles (SPIOs) 53, 54, 55; complete protocols are presented in 44, 54. For *i.c*. injection, hCE1m6‐NSCs were suspended at 0.1–2.0 × 10^5^ cells in 2 µl Ca^2+^/Mg^2+^‐containing PBS with heparin (10 µg/ml) and injected stereotactically under ketamine/xylazine anesthesia. For *i.v*. tail vein injection, hCE1m6‐NSCs were suspended at 0.1 × 10^5^–2.0 × 10^6^ cells in 200 µl Ca^2+^/Mg^2+^‐containing PBS with heparin (10 µg/ml). Previous studies have shown that HPF labeling does not affect cell viability, growth kinetics, or tumor tropism in vitro 44, 54.

### Histological Procedures

Mice were euthanized on day 3 after NSC administration (7 days after U251 cell engraftment). Brains (nonperfused) were harvested and fixed in 50 ml of 4% paraformaldehyde (PFA) in PBS for 72–96 hours while cooled in an ice and water slush on a rocking platform. Brains were then rinsed with reagent‐grade water, placed in 70% ethanol, and stored at room temperature. Conventional paraffin blocks were prepared and 10 µm‐thick horizontal serial sections through the tumor were cut in the City of Hope Pathology Core. Typically each tumor extended 2.5–3.5 mm vertically, and after trimming approximately 500 µm from the dorsal brain surface, 120–180 slides were collected, each carrying two 10 µm‐thick sections, through the entire tumor. Each slide was bar coded for identification by experiment, brain and section number. Slides were considered in groups of ten. The first slide in each group was processed for hematoxylin and eosin (H+E) staining, the adjacent (2nd) slide was immunochemically stained for eGFP to identify U251 tumor cells, and the subsequent (3rd) slide was processed for Prussian blue chemistry to identify Feraheme‐labeled hCE1m6‐NSCs.

For staining, paraffin sections were baked at 57°C for 3 hours, then deparaffinized and rehydrated through a series of xylene and graded alcohol solutions. Prussian blue staining was performed using the Accustain iron stain kit (Sigma‐Aldrich; St. Louis, MO; www.sigmaaldrich.com), according to the manufacturer's protocol. Prussian blue sections were counterstained with Pararosaniline (Sigma P3750). Adjacent sections were stained with anti‐eGFP antibody (ab290, 1:500 dilution; Abcam; Cambridge, MA; www.abcam.com) and processed for immunoperoxidase‐3,3′‐diaminobenzidine (DAB) using Vectastain ABC Elite and peroxidase substrate kits (Vector Laboratories; Burlingame, CA; vectorlabs.com) following the manufacturer's instructions. DAB‐reacted sections were counterstained with hematoxylin.

### Image Acquisition

Each slide was scanned at high resolution (approximately 15,000 × 15,000 square pixels at 1 µm per pixel) and 24‐bit RGB images of entire brain sections were acquired using an ACIS II scanning microscope (Chromavision; Dako; Santa Clara, CA; www.agilent.com/en-us/dako-products). These were converted to TIFF or JPEG format using custom scripts (MacroExpress; Insight Software; Kaysville, UT; www.wintools.com).

### Image Segmentation and Determinations of Tumor Volume, hCE1m6‐NSC Distribution, and Estimated Tumor Coverage by a Diffusible Chemotherapeutic

The scheme for image processing is shown in Figure 2. Images of a pair of chromagen‐stained brain slices as generated by the ACIS II scanning microscope are shown in Figure 2A*1* and 2A*2*, with hCE1m6‐F3 NSCs visualized by Prussian blue (upper panel) and U251 tumor cells immunostained with anti‐eGFP/DAB (lower panel). These images were segmented into channels representing NSCs or tumor cells by color deconvolution as illustrated in Figure 2B with pixels corresponding to hCE1m6‐F3 NSCs colored red and U251 tumor cells colored dark green. This was done using the algorithm published by Ruifrok and Johnston 56 and incorporated into the “Colour Deconvolution” plugin (http://fiji.sc/Colour_Deconvolution; http://www.mecourse.com/landinig/software/cdeconv/cdeconv.html) for Fiji 57 (http://fiji.sc/Fiji). Numbers of hCE1m6‐F3 NSCs were determined for each 10 µm‐thick section by manual counting of individually resolved cells, or by dividing the total number of Prussian blue‐marked pixels by the average number of pixels per NSC. Total numbers of hCE1m6‐F3 NSCs in a tumor were determined by extrapolating over the 200 µm separating each group of ten slides, and then summing these values.

**Figure 1:**
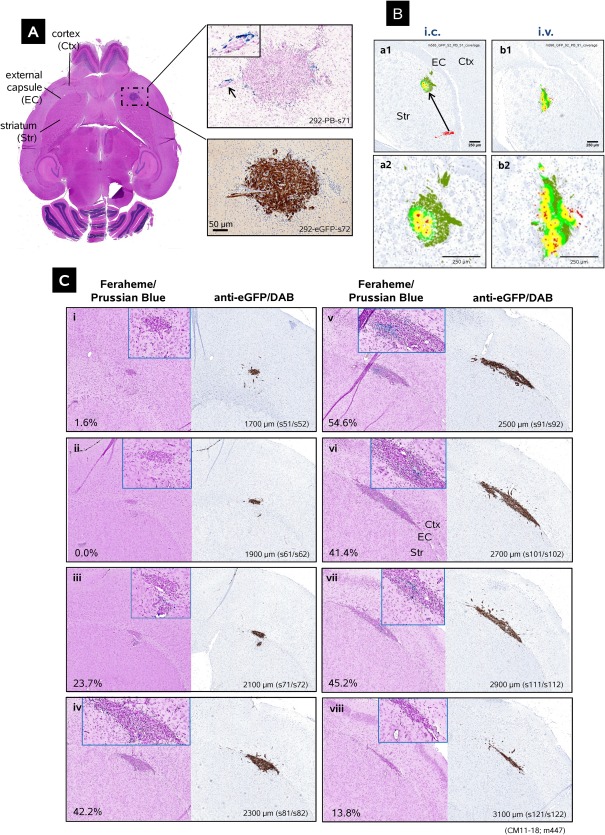
Distributions of iron‐labeled hCE1m6‐F3 neural stem cells (NSCs) administered *i.c*. into the frontal lobe of U251 glioma‐bearing mice. **(A)**: Low‐power image of a hematoxylin‐eosin (H+E)‐stained mouse brain section. (right upper) expanded area from A showing Prussian blue‐stained hCE1m6‐NSCs (pararosaniline counterstain) at the tumor mass and an invasive tumor nodule (inset); (right lower) DAB‐visualized (brown) eGFP‐expressing U251 glioma cells of the same tumor mass from the next slide (hematoxylin counterstain). Scale bar = 50 µm. **(B)**: Distribution of hCE1m6.NSCs at tumor sites after *i.c*. (*a1*, *a2*) or *i.v*. (*b1*, *b2*) administration. NSCs are marked in red and tumor cells are colored according to their distance from the NSCs (yellow for ≤50 µm, light green for ≤100 µm, and dark green for >100 µm). For *i.c*. NSC administration, the arrow indicates apparent migration of a subset of NSCs from the injection site to the tumor. Scale bar = 250 µm. **(C)**: **(Ci–Cviii)** Paired images of consecutive sections (feraheme‐Prussian blue and eGFP‐DAB) showing distributions of hCE1m6‐F3 NSCs within and around an engrafted U251 tumor at increasing depths below the pial surface (indicated) and thus in different anatomical locations within the host brain. Note the disseminating U251 glioma cells in many sections, and lateral expansion in the external capsule along with the presence of hCE1m6‐F3 NSCs at greater depths. Positions of Ctx, EC, and Str are indicated in panels A, B*a1* and C*vi*. Estimated per cent tumor coverage by a secreted therapeutic (50 μm effective radius) determined as described (Fig. 2 and text) is also indicated. Images in A and C show tumors at 7 days engraftment and four days after NSC administration; tumors in B are at 14 days engraftment and 4 days post‐NSC injection. Abbreviations: Ctx, cortex; EC, external capsule; Str, striatum.

**Figure 2:**
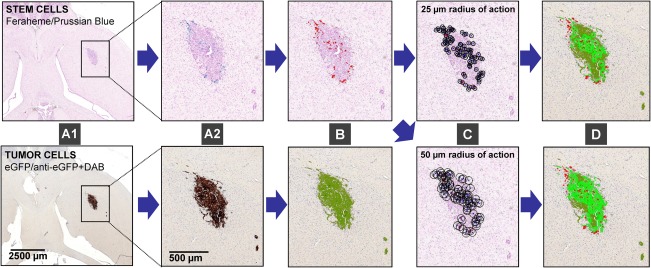
Distribution hCE1m6‐F3 neural stem cells (NSCs) and representation of predicted tumor coverage by a diffusing chemotherapeutic. **(A1**, **A2)**: Consecutive brain sections stained for hCE1m6‐F3 NSCs (above, Prussian blue for feraheme) and U251 glioma cells (below, DAB immunostaining of eGFP). (**B**): Segmentation of cells of interest by color deconvolution (see Methods) with hCE1m6.F3NSCs shown in red and U251 glioma cells in dark green. Small tumor nodules were included in analyses by virtue of this segmentation method, which was based on expression of human nestin by all tumor cells rather than by delineation of the tumor mass which will be limited by tumor morphology. **(C)**: Computed radii (circle 25 µm above and 50 µm below) centered on each cell. **(D)**: Estimated tumor coverage by a secreted drug (light green) centered on each pixel at the edge of each stem cell (red) for a particular radius of action from each stem cell.

Figure 2C gives an indication of the spatial domain of each hCE1m6‐F3 NSC using circles of radius with circles of radius 25 or 50 µm drawn from the centroid of each cell. Figure 2D illustrates tumor coverage of a secreted therapeutic such as CE estimated by drawing a radius of the desired length from the edge of each Prussian blue‐marked area, and determining the numbers of tumor cell (eGFP‐positive) pixels within this area using Cell Meter software developed for MatLab (MathWorks; Natick, MA; www.mathworks.com) (http://www.pedimg.org/cellmeter/software.html; http://www.youtube.com/watch?v=MDbEHrdTolk). In Figure 2D, the entire tumor is shown in green, with each pixel within the estimated radius of action shown in lighter green.

Tumor volume was quantified according to the Cavalieri principle by extrapolating the numbers of DAB‐stained pixels over the 200 µm separating each set of 10 slides. For three‐dimensional reconstruction, images of each section were manually aligned with ventricles and olfactory bulbs as landmarks using Reconstruct 58 (http://synapses.clm.utexas.edu/tools/reconstruct/reconstruct.stm).

*X*, *Y* positions of NSCs within tumors were obtained using CellProfiler v2.2 (Broad Institute; Cambridge, MA; http://cellprofiler.org/) 59, and pair‐wise distances between any two NSCs (*i, j*) determined from the Euclidian relation $d_{i,j}=\sqrt{{d(x_{j}-x_{i})}^{2}+{d(y_{j}-y_{i})}^{2}}$ (see Fig. 7 legend).

Estimations of the diffusion of carboxylesterase (CE) in brain were made as described by Wolak and Thorne 60, assuming a molecular weight of 60,000 61, 62 and a diffusion coefficient (*D*) in free solution of 4.3 × 10^−7^ cm^2^ per second 62. D*, the diffusion coefficient adjusted for the tortuosity of brain extracellular space (λ), was calculated using the relation D* = *D*/λ^2^, where λ was 2.25, a value characteristic of high molecular weight molecules in brain tissue 63. D* for CE in brain was thus 8.5 × 10^−8^ cm^2^ per second. Relative CE concentration (*C*/*C*_0_) at distance *x* from a nondepleting source assuming essentially no efflux or degradation (*k*_e_ = ln(2)/*t*_1/2_) was then calculated from the relation *C*/*C*_0_ = exp{–*x*(*k*_e_/D*)^1/2^} 64.

Data were compiled and analyzed in Excel (Microsoft; Bellevue, WA; office.microsoft.com/excel) and are presented using SigmaPlot 13 (Systat; San Jose, CA; systatsoftware.com). Statistical tests were made using Prism 7 (GraphPad; La Jolla, CA; www.graphpad.com) or Excel. For correlations between numbers of NSCs administered and other parameters, *r* ^2^ and *p* are reported for linear regressions. For correlations between other not predetermined parameters, *r* (the Spearman correlation coefficient) and *p* are reported. Other comparisons were evaluated by *t* test as noted.

## Results

Presented here are the results of an analysis of a cohort of orthotopic U251 tumor xenografts, 14 across two independent experiments with *i.c*. NSC administration near the site of the engrafted tumor, and 22 across two independent experiments with *i.v*. NSC administration. Tumors were studied at 1 week following implantation, with hCE1m6‐NSCs delivered on day 4 and animals sacrificed 3 days later.

### Tumor Architecture and NSC Infiltration

Figure 1A presents a representative H+E stained horizontal section of U251 tumor. An area of dense tumor nuclei is evident in hematoxylin staining, centered on the external capsule and extending into the striatum and cortex. The lower right panel shows U251 cells in the adjacent slide identified by eGFP antibody and DAB immunochemistry, and the upper right panel shows feraheme‐loaded NSCs in the preceding slide stained with Prussian blue. In this brain, NSCs were found around the edge of the main tumor mass, and along an invading nodule (upper panel, black arrow).

NSCs were found at the external tumor margins and penetrating the tumor interior, as illustrated by the two brain sections in Figure 1B in which tumor cells engrafted and formed masses within the striatum after *i.c*. or *i.v*. administration. Here (in greater detail in Fig. 2 *a2*, 2*b2*), tumor cells are shown in dark green, light green, or yellow depending on their diffusion proximity to the hCE1m6‐F3 NSCs highlighted in red. The distribution of NSCs both at the tumor margins and within the tumor masses did not appear to differ between *i.c*. and *i.v*. administration routes. For other examples, see 54.

Not all injected NSCs showed tumor‐directed mobility. For the brain shown in Figure 1B*a1*, hCE1m6‐F3 NSCs were administered *i.c*. just caudal lateral to the tumor. The arrow indicates the migration of a portion of the NSCs from the injection site in the striatum to the tumor mass. When tumors were present, we did not observe migration of NSCs toward other brain structures.

Within a given brain, tumor morphology and the distributions of hCE1m6‐NSCs could vary greatly with depth from the pial surface and thus position in the brain. The example presented in Figure 1C shows paired images of Prussian blue stained NSCs that were injected *i.v*. just caudal‐lateral to the eGFP‐DAB stained tumor, taken at increasing depths beginning approximately 1,700 µm below the dorsal brain surface and proceeding in 200 µm increments to 3,100 µm. Because of the arrangement of brain structures, in more dorsal sections the tumor was found engrafted in cortex, as in panel C*i*. At greater depths the tumor engrafted at the border of the striatum and external capsule, and, as was commonly observed, disseminated laterally following the arc of the external capsule (panels C*iii*–Cv*iii*). Tumor cells also migrated into the striatum and cortex at all depths (panels C*i*–*viii*). Viewing sections from the entire tumor, observations such as those in Figure 1C suggest the presence of a microenvironment in the white matter of the external capsule particularly advantageous for both tumor cells and NSCs.

Annotations to Figure 1C indicate the estimated percentage of total tumor area that would be exposed to a therapeutic secreted by the NSCs (assuming a 50 µm radius of action as described in greater detail below) at different vertical locations within the tumor. As seen here, the most extensive tumor coverage was seen for larger tumor cross sections more central to the vertical span of the engrafted tumor (see also Fig. 5B and 65).

### Quantitative Analyses

The procedures for quantifying hCE1m6‐NSC numbers and estimating tumor areas exposed to secreted CE are described in the Methods and shown schematically in Figure 2. Images of a pair of brain slices show hCE1m6‐F3 NSCs visualized by Prussian blue (upper panels of Fig. 2A1, 2A2) and U251 tumor cells immunostained with anti‐eGFP/DAB (lower panels). Deconvolution yielded color channels representing NSCs in red pixels and tumor cells in green pixels (Fig. 2B). This process yields the numbers and locations of hCE1m6‐F3 NSCs and quantitative measures of tumor area. The percentage of total tumor area in each brain slice that would be exposed to a diffusing therapeutic secreted by the NSCs can then be calculated (Fig. 2C) and visualized (Fig. 2D) in light green for diffusion radii of 25 and 50 µm around the border of each red NSC.

We focused on the localization of NSCs to tumor sites, and on variables influencing tumor coverage that could be achieved by a diffusing therapeutic. This therapeutic design incorporates NSC delivery of an enzyme expected to diffuse in and around the tumor site and catalyze formation of a chemotherapeutic from a systemically administered prodrug 50.

**Figure 3:**
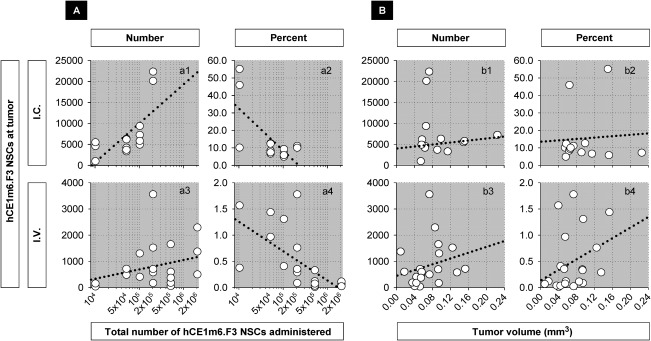
Migration of hCE1m6‐F3 NSCs to tumor sites after *i.c*. or *i.v*. administration. Numbers of NSCs counted at tumor sites are shown in relation to **(A)** the numbers administered and **(B)** tumor volume. Doses of hCE1m6‐F3 NSCs were 10–200 × 10^3^ cells for *i.c*. and 0.01–2.0 × 10^6^ cells for *i.v*. routes. In this and subsequent figures, each dot represents data from an individual mouse. The dotted lines are linear regressions given to provide a visual reference to trends in the data, and have no other significance. In some graphs X‐axes are log_10_ to provide for visual expansion of the data. **(A)** Comparison to total numbers of NSCs administered (a1, a3) Number of NSCs at tumor in relation to numbers of NSCs administered *r* ^2^ = 0.81, *p* < .0001 for *i.c*.; *r* ^2^ = 0.06, *p* = .28 for *i.v*. (a2, a4) Percentage of NSCs at tumor in relation to numbers of NSCs administered *r* ^2^ = 0.20, *p* = .12 for *i.c*.; *r* ^2^ = 0.22, *p* = .03 for *i.v*. **(B)** Comparison to total tumor volume (b1, b3) Number of NSCs at tumor in relation to tumor volume *r* = 0.26, *p* = .37 for *i.c*.; *r* = 0.43, *p* = .05 for *i.v*. (b2, b4) Percentage of total administered NSCs found at tumor in relation to tumor volume *r* = 0.35, *p* = .22 for *i.c*.; *r* = 0.34, *p* = .12 for *i.v*. Abbreviation: NSCs, neural stem cells.

Approaching first the NSCs distributed at tumor sites, we observed, not surprisingly, that after *i.c*. injection, numbers of tumor‐associated NSCs scaled with the number of cells administered (Fig. 3 *a1*, *p* < .0001), while there was considerably more unpredictability in number of NSCs at tumor sites after *i.v*. injection (Fig. 3 *a3*, *p* = .28). This might be expected given that the *i.c*. route deposits the NSCs close to the tumor site while NSCs administered *i.v*. must circulate prior to responding to as yet not well‐defined vascular cues 37, 66, and then extravasate into the tumor parenchyma.

The percentages of total injected hCE1m6‐F3 NSCs ultimately recovered at tumor sites was always larger for *i.c*. administrations, as high as 50%–60% for small NSC doses and around 10% for larger doses (Fig. 3 *a2*). For *i.v*. administered hCE1m6‐F3 NSCs, around 1.5% of NSCs were recovered for smaller doses and 0.3% or less for larger doses (Fig. 3 *a4*). In similar models, other investigators 65, 67 have reported recovery of comparable percentages of cells after *i.c*. NSC injections.

**Figure 4:**
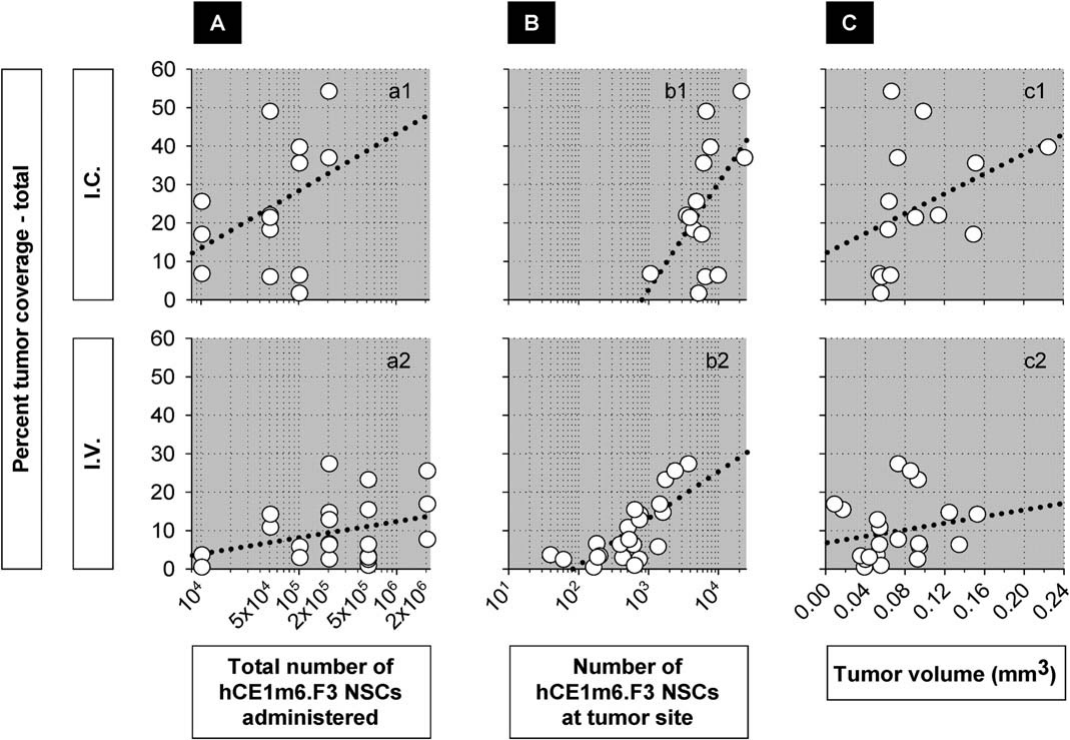
Percentage of tumor coverage by an NSC‐secreted therapeutic. Coverage is estimated (see Methods) assuming a 50 µm radius of action for delivery *i.v*. or *i.c*. **(A)**: Percentage tumor coverage in relation to numbers of NSCs administered (a1) *r* ^2^ = 0.26, *p* = .07 for *i.c*.; (a2) *r* ^2^ = 0.12, *p* = .12 for *i.v*. **(B)**: Percentage tumor coverage in relation to numbers of NSCs at the tumor site (b1) *r* = 0.42, *p* = .14 for *i.c*.; (b2) *r* = 0.73, *p* = 1.07 × 10^−4^ for *i.v*. **(C)**: Percentage tumor coverage in relation to tumor volume (c1) *r* = 0.60, *p* = .03 for *i.c*.; (c2) *r* = 0.24, *p* = .23 for *i.v*. Abbreviation: NSCs, neural stem cells.

Another parameter potentially related to NSC attraction is tumor volume, and we examined possible correlations between tumor volume and the numbers and percentages of hCE1m6‐NSCs reaching tumor sites. For both *i.c*. and *i.v*. injections, we observed that hCE1m6‐F3 NSCs could be found associated with very small tumor masses (Fig. 3 *b1*, 3*b3*). Examined more quantitatively, the numbers of NSCs observed at tumor sites were not significantly related to tumor size after *i.c*. injection (Fig. 3 *b1*, *p* = .37), but for *i.v*. injection tumor size did appear to influence NSC attraction (Fig. 3 *b3*, *p* = .05). For the percentages of total administered NSCs at tumor sites, tumor size did not appear to be a factor for i.c. injections (Fig. 3 *b2*, *p* = .22), and not strongly for i.v. injections (Fig. 3 *b4*, *p* = .12) (see also 65).

Because the ultimate therapeutic efficacy of this NSC‐based therapy will depend on the delivery of a diffusing prodrug‐activating enzyme (“bystander effect” 68), we next undertook an analysis of the percentage of total tumor volume that would be exposed to a soluble therapeutic in relation to these same three parameters: total number of NSCs administered, the numbers of NSCs subsequently localized to tumor sites, and total tumor volume.

We calculated percentage tumor coverage for radii of action of both 25 and 50 µm for an enzyme originating in the NSCs, distances representing 3–4 or 6–8 diameters of 8–10 µm cells. First, calculations of the diffusion radius of carboxylesterase (CE) incorporating its molecular weight, free diffusion coefficient in solution, and tortuosity of the brain and brain tumor extracellular space (see Methods) indicate that at steady state, 50% of initial concentration will be seen at 25 µm and 24% at 50 µm distance from a secreting source. That CE‐driven therapeutic activity will be seen at these distances is suggested by results of in vitro experiments 69 showing both efficient CE‐catalyzed conversion of CPT‐11 to SN‐38, and high sensitivity of glioma cells to SN‐38 (IC_50_ of 9 to 152 nM against patient‐derived and established glioma cell lines). In addition, results from in vivo models 70 indicate that significant tumor regression can be observed when only 1 in 50 cells (2%) express an activating enzyme, in this case cytosine deaminase (CD) to locally form a chemotherapeutic (5‐fluorouracil, 5‐FU) from a systemically administered prodrug (5‐fluorocytosine, 5‐FC). For this system Lin et al. 65 calculated an estimated killing radius of 23 µm.

In our experiments, total tumor coverage, estimated assuming a 50 µm effective radius, appeared to depend on the numbers of hCE1m6‐NSCs administered, and could reach greater than 50% for the highest dose delivered *i.c*. (2.0 × 10^5^) (Fig. 4 *a1*, *p* = .07) (see also 55, 65), and above 25% for *i.v*. delivery of up to 10 times as many cells (2.0 × 10^6^) (Fig. 4 *a2*, *p* = .12). This pattern of half as much coverage for 10 times the number of hCE1m6‐F3 NSCs administered *i.c*. versus *i.v*. appeared consistently across all NSC doses examined. At the tumor site, percentage tumor coverage also appeared greater for higher numbers of hCE1m6‐NSCs after *i.c*. delivery (Fig. 4 *b1*, *p* = .14) and was quite significant for *i.v*. delivery (Fig. 4 *b2*, *p* = 1.07 × 10^−4^). There was also a trend toward greater percentage coverage of larger tumors (Fig. 4 *c1*, *p* = .03 for *i.c*.) (Fig. 4 *c2*, *p* = .23 for *i.v*.). Percent tumor coverage was most highly correlated to tumor volume for *i.c*. delivery, perhaps reflecting the relative simplicity of the NSC path.

**Figure 5:**
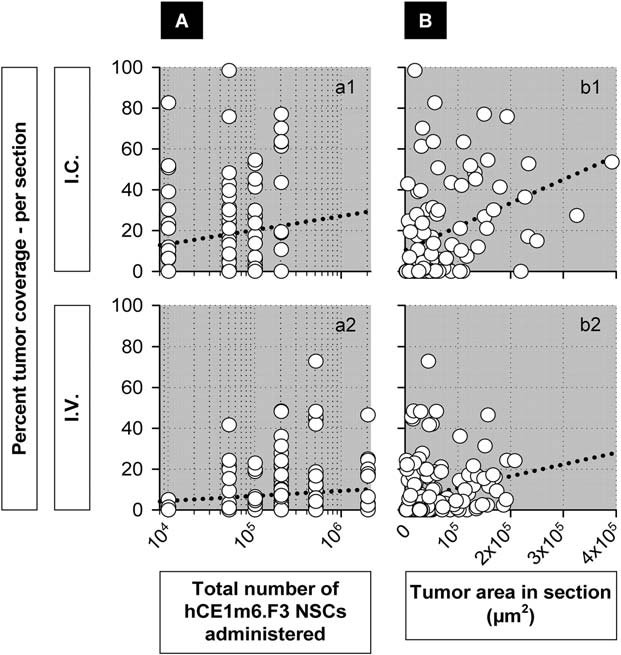
Percentage tumor coverage evaluated on individual brain sections. All brain sections containing engrafted tumor cells in the cohort of 14 *i.c*.‐ and 22 *i.v*.‐injected brains were evaluated without regard to the presence or absence of neural stem cells (NSCs); *n* = 101 sections for *i.c*. and 161 for *i.v*. **(A)**: Percentage tumor coverage in relation to numbers of NSCs administered (a1) *r* ^2^ = 0.03, *p* = .07 for *i.c*.; (a2) *r* ^2^ = 0.003, *p* = .49 for *i.v*. **(B)**: Percentage tumor coverage in relation to tumor area in each section (b1) *r* = 0.44, *p* = 3.29 × 10^−6^ for *i.c*.; (b2) *r* = 0.41, *p* = 9.90 × 10^−8^ for *i.v*. Abbreviation: NSCs, neural stem cells.

To explore this point further, we analyzed distributions of hCE1m6‐F3 NSCs on a per section basis, taking measurements from one section of each slide at every depth in which both tumor cells and NSCs were present. For both *i.c*. and *i.v*. administrations, percent tumor coverage was highly correlated with tumor area within each section (Fig. 5 *b1*, *p* = 3.29 × 10^−6^ for *i.c*) (Fig. 5 *b2*, *p* = 9.90 × 10^−8^ for *i.v*.), and much less so for NSC dose (Fig. 5 *a1*, *p* = .07 for *i.c*.) (Fig. 5 *a2*, *p* = .49 for *i.v*.).

**Figure 6:**
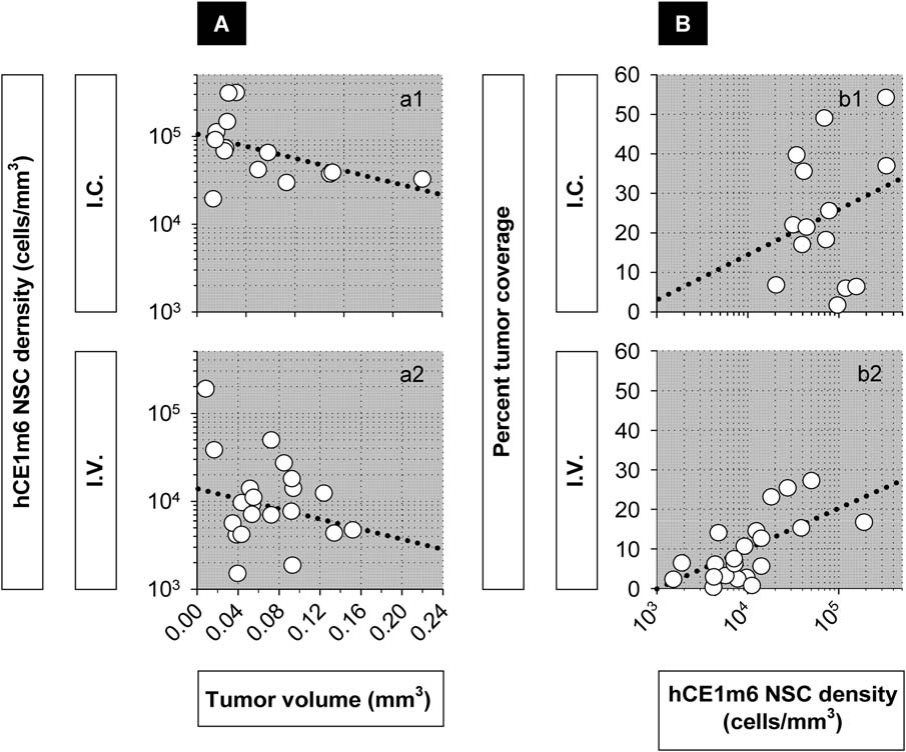
Neural stem cell (NSC) density and its influence on tumor coverage. **(A)**: Density of hCE1m6‐F3 NSCs in relation to tumor volume (a1) *r* = −0.35, *p* = .22 for *i.c*.; (a2) *r* = −0.002, *p* = .99 for *i.v*. **(B)**: Percent tumor coverage in relation to density of hCE1m6‐F3 NSCs (b1) *r* = 0.02, *p* = .95 for *i.c*.; (b2) *r* = 0.66, *p* = .001 for *i.v*. Average percent tumor coverage for *i.c*. delivery is 23.8 ± 14.5% for the density range 1.9 × 10^4^–9.2 × 10^4^ NSCs/mm^3^, and for *i.v*. delivery is 15.8 ± 9.4% for the density range 1.1 × 10^4^–5.0 × 10^4^. Within this range of overlapping densities, *p* = .18 (two‐tailed two‐sample *t* test, unequal variance), indicating that there was no statistically significant difference in average percent coverage between the two routes of NSC administration. Abbreviation: NSCs, neural stem cells.

We also investigated whether NSC “history” during migration from *i.c*. or *i.v*. administration sites exerted influence on behavior of NSCs after their arrival at tumor site. NSC density (numbers of NSCs present per unit of tumor volume) appeared to be negatively related to tumor size for both *i.c*. and *i.v*. administration (Fig. 6 *a1*, 6*a2*). Unexpectedly, the percent tumor coverage did not appear tightly related to the density of hCE1m6‐F3 NSCs for *i.c*. administration (Fig. 6 *b1 p* =.95 for *i.c*), but appeared more so for *i.v*. administration (Fig. 6 *b2*, *p* = .001 for *i.v*). For the range of hCE1m6‐F3 NSC densities spanning approximately 10^4^–10^5^ NSCs/mm^3^, there was no statistically significant difference in percent coverage for NSCs delivered *i.c*. and *i.v*. Of note, it may be possible to increase the NSC density at tumor sites by the i.v. route using repeat administrations.

**Figure 7:**
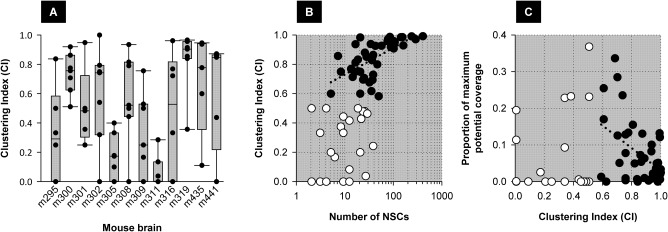
Nonuniform distribution of hCE1m6‐F3 neural stem cells (NSCs) and consequences for the efficiency of tumor coverage by a secreted therapeutic. hCE1m6‐F3 NSCs were often found in clusters and close to the tumor edge. As a quantitative measure of clustering, the Euclidean distance between each pair of NSCs was calculated from the *x*, *y* coordinates of each NSC (see Methods). The clustering index (*CI*) is defined for each section as 1 – [(number of clusters)/(total number of NSCs)], such that a higher *CI* indicates greater clustering. **(A)**: Clustering indices (*CI*s) for each section in a cohort of 73 individual tumor‐bearing brain sections from 12 brains with *i.c*.‐administered NSCs. Sections contained between 2 and 390 NSCs. Shown for each brain are individual section *CI*s, and box plots where the box spans the 25th to the 75th percentile, the error bars indicate the 10th and 90th percentiles, and the horizontal bar marks the median, with *CI* values for each section are superimposed. **(B)**: Relationship between the number of NSCs present at the tumor and the *CI*. Open symbols mark *CI* ≤ 0.5 for which there was considerable scatter. Filled symbols mark *CI* > 0.5 for which the *CI* appears to increase with the number of NSCs. **(C)**: Relationship between the tumor coverage estimated for each section assuming a therapeutic radius of 50 µm and the theoretical maximum assuming homogeneous distribution of NSCs through the tumor. Symbols are as in (B); greater clustering (higher *CI*) appears associated with reduced efficiency. Abbreviation: NSCs, neural stem cells.

The effective efficiency of tumor coverage by a diffusing therapeutic, as compared to the theoretical maximum obtainable assuming homogeneous NSC distribution throughout a tumor mass, will depend on the distances between NSCs (as overlap of radii of diffusion may lead to redundancy) and on NSC proximity to the tumor edge (as radii of diffusion may extend into the brain parenchyma). Both of these situations can be seen in Figures 1B and 2D. We therefore assessed the potential contribution of NSC clustering on tumor coverage by using pair‐wise distances between NSCs in individual sections *i.c*.‐injected with NSCs to determine a clustering index (*CI*) for each section based on a clustering radius of 50 µm, where *CI* = 1 would indicate that all NSCs were in a single cluster and *CI* = 0 would indicate complete dispersion. *CI*s were quite variable between brains and between sections within individual brains, as illustrated in Figure 7A, and with considerable scatter were related to the numbers of NSCs within each tumor section (particularly for larger numbers of NSCs). The proportion of the theoretical maximum tumor coverage achieved in each section also appeared to decrease with greater NSC clustering (higher *CI*) (Fig. 7C).

## Discussion and Conclusion

In summary, in this orthotopic xenograft model of human glioma, we have demonstrated that quantitative analyses of NSC homing and biodistribution in relation to glioma masses can be performed using standard formalin‐fixed paraffin‐embedded (FFPE) brain sections and freely‐available software tools running on personal computers of reasonable power.

We conclude that hCE1m6‐F3 NSC therapies are “well behaved” in that administering higher doses tends to yield increased numbers of NSCs at tumor sites (Fig. 3A) and greater estimated tumor coverage by a diffusing therapeutic (Fig. 4A). Further, while at least 10‐times as many NSCs must be administered *i.v*. as compared to achieve to *i.c*. to achieve a given NSC density, our findings suggest that NSC behaviors at tumors are equivalent for the two administration routes, and that similar NSC densities, once achieved, yield similar tumor coverage.

At the same time, the process of NSC homing appears to limited by one or more rate‐limiting process active during administration and/or migration, in that the percentage of administered NSCs ultimately localized to tumor sites falls with injection of larger numbers of cells for both *i.c*. and *i.v*. routes (Fig. 3A). This finding was unexpected, as all things being equal, one might expect a constant percentage of NSCs to arrive at tumor sites independent of the numbers of cells injected. Therefore, the more efficient propagation of NSCs to tumor sites seen with lower numbers of NSCs administered by both *i.c*. and *i.v*. routes implies activity of additional process possibly related to absolute numbers of NSCs, to the concentration of NSCs in the injection vehicle, and/or to the densities of NSCs at the injection sites, that limit NSC survival. Alternatively, the ability of tumor masses to absorb NSCs may be limited, leading to saturation as larger numbers of NSCs are injected. These possibilities will be further investigated.

More efficient delivery of small number of NSCs has been noted by other investigators 65, 67, and this remains a bottleneck in implementation of NSC‐based therapies. Enhancements in NSC delivery (such as inclusion in biocompatible matrices 71, 72) might improve NSC survival and therapeutic efficiency, while breaking NSC administrations into multiple injections might reduce tumor saturation if present, and could have implications for design of NSC delivery schedules (e.g., single bolus vs. multiple doses).

We also find that larger tumors, and larger regions of individual tumors, tend to attract greater numbers of NSCs, and there is a trend toward higher NSC densities and greater estimated therapeutic coverage in larger tumors. This pattern could be linked to higher levels of inflammatory cytokines associated with greater numbers of tumor cells and/or tumor‐associated monocytes and immune cells, or to the more extensive brain disruption or injury that would be expected with a larger tumor. A similar association of NSC accumulation with areas of higher tumor cell density was reported by Lin et al. 65 (but see 55).

Finally, we observed that the degree to which NSCs cluster together also impacts the efficiency of tumor coverage by a secreted therapeutic as compared to the theoretical maximum, raising the possibility of manipulating the (presently unknown) factors that influence NSC clustering.

The analysis outlined here provides a framework by which investigators may rationally evaluate NSC migration to, and integration into, brain tumors, and thereby increase our understanding of this mode of therapy delivery. However, despite use of a standardized model, considerable scatter was observed in the data that may arise from multiple sources, including variation in tumor engraftment depending on position in the brain, and variable penetration of NSCs into tumor masses, as well as details of NSC administration. Identifying and these uncontrolled variables in therapy implementation and relating them to details of the administered product and to tumor cellular composition and physiology will enhance further development of stem cell‐based therapies.

## Authors Contributions

M.E.B.: Conception and design, data analysis and interpretation, manuscript writing, final approval of manuscript; K.H.: Conception and design, collection and/or assembly of data, data analysis and interpretation; Y.T.: Data analysis and interpretation; S.A.H.: Conception and design, collection and/or assembly of data, data analysis and interpretation; M.M.: Provision of study material or patients; S.A.: Provision of study material or patients, collection and/or assembly of data; R.T.: Provision of study material or patients, collection and/or assembly of data; M.G.: Data analysis and interpretation, manuscript writing; A.A.: Collection and/or assembly of data, data analysis and interpretation; R.A.M.: Collection and/or assembly of data, data analysis and interpretation; L.G.: Data analysis and interpretation; R.C.R.: Data analysis and interpretation; J.G.: Data analysis and interpretation; C.E.B.: Provision of study material or patients, data analysis and interpretation; L.G.: Administrative support, data analysis and interpretation; K.S.A.: Conception and design, financial support, provision of study material or patients, manuscript writing, final approval of manuscript.

## Disclosure of Potential Conflicts of Interest

A.J.A. and K.S.A. are share‐holders, directors and officers of TheraBiologics, a clinical stage biopharmaceutical company focused on the development of stem cell‐mediated cancer therapy. R.A.M. is a director of TheraBiologics. L.G. is employed by Infosphere Clinical Research partner. C.B. indicates Spouse has employment at Xencor, Inc., CAR T cell patents licensed by Mustang Bio, and Scientific Advisory Board for Mustang Bio. The remaining authors have indicated no potential conflicts of interest.
